# Human Amniotic Membrane-Derived Mesenchymal Stem Cell-Conditioned Saline as an Injectable Formulation Improves Ovarian Antioxidant Status and Preimplantation Embryo Development

**DOI:** 10.3390/biomedicines14071522

**Published:** 2026-07-07

**Authors:** Kihae Ra, Eun Young Kim, Sung Keun Kang, Geon A Kim, Se Chang Park

**Affiliations:** 1 College of Veterinary Medicine and Research Institute for Veterinary Science, Seoul National University, Seoul 08826, Republic of Korea; 2 RNL Regeneration Medicine Research Institute Co., Ltd., Seoul 08506, Republic of Korea; 3 Department of Biomedical Laboratory Science, College of Health Science, Eulji University, Uijeongbu 11759, Republic of Korea; 4 Laboratory of Aquatic Biomedicine, College of Veterinary Medicine and Research Institute for Veterinary Science, Seoul National University, Seoul 08826, Republic of Korea

**Keywords:** amniotic membrane, mesenchymal stem cell, cell-free therapy, assisted reproductive technology, in vitro fertilization, female reproduction, oxidative stress

## Abstract

**Background/Objectives**: Oxidative stress is a major cause of impaired oocyte quality and early embryo development, a challenge that still needs to be addressed in assisted reproduction. Mesenchymal stem cell secretomes have been investigated as cell-free therapeutics with antioxidant activity and relevant anti-apoptotic effects. This study aimed to evaluate the effects of human amniotic membrane-derived mesenchymal stem cell-conditioned saline (AMSC-CS) as an injectable formulation on oxidative stress–related markers in ovarian tissue and preimplantation developmental outcomes. **Methods**: AMSC-CS was administered intravenously to female mice in a dose-dependent manner. Safety assessments were conducted to evaluate systemic and target organ toxicity within the dosage range. In vitro fertilization (IVF) outcomes and oxidative status in ovaries, oocytes, and embryos were evaluated following treatment with low, medium, and high doses of AMSC-CS (1, 3, and 5 μL/g). **Results**: As an injectable formulation, the safety assessments did not reveal systemic or target organ toxicity of AMSC-CS within the dosage range. Medium-to-high doses of AMSC-CS improved the expression of folliculogenesis-related genes and decreased oxidative stress and apoptosis signaling in ovarian tissue. At the high dose, AMSC-CS promoted preimplantation embryo development to the blastocyst and hatched blastocyst stages, along with improved blastocyst quality and reduced oxidative stress in oocytes and blastocysts. **Conclusions**: These findings suggest that AMSC-CS at medium-to-high doses, as an injectable formulation with antioxidant activity, may be a promising adjunct for assisted reproductive technologies.

## 1. Introduction

Oocyte quality and the developmental competence of early embryos are key factors that determine the success of implantation and pregnancy, and are particularly important conditions in assisted reproduction [1]. Oxidative status in the ovarian microenvironment has a strong influence on the quality of oocytes and embryos [2]. Excessive reactive oxygen species (ROS) can damage mitochondria, disturb cellular homeostasis in oocytes and embryos, and activate apoptotic signaling, which reduces their developmental potential [3]. Conversely, enhancing the antioxidant defense system by regulating transcription factors and antioxidants, such as superoxide dismutase (SOD), glutathione peroxidase (GPx), and catalase (CAT), helps to limit oxidative damage and inhibit downstream caspase activation and Bax/Bcl2-mediated apoptosis, thereby supporting the developmental process of oocytes and embryos [4,5,6]. As stress response transcription factors, FOXO1 and FOXO3, members of the FOXO family, are highly expressed in ovarian follicles and have been known to regulate granulosa cell survival and follicle growth, support sustained reproductive capacity, and contribute to oxidative stress resistance by modulating antioxidant enzymes [7,8,9]. SOD, GPx, and CAT constitute a major enzymatic defense system that detoxifies ROS and limits oxidative stress in ovarian tissues and oocytes [10,11]. SOD converts superoxide anions into hydrogen peroxide, which is then reduced to water by GPx and CAT, helping to alleviate the overall oxidative burden and maintain mitochondrial integrity. Consequently, decreased activity of SOD, GPx, and CAT in the ovaries leads to increased oxidative stress, accelerated follicular atresia, and impaired ovarian function [10,12]. Furthermore, SOD, GPx, and CAT contribute to protecting oocytes and preimplantation embryos from ROS-induced damage [13]. If antioxidant defense is insufficient, excessive ROS can increase the Bax/Bcl2 ratio and mitochondrial outer membrane permeability, potentially triggering cytochrome c release and caspase activation [14]. Conversely, adequate SOD, GPx, and CAT activity can mitigate ROS accumulation, prevent pathological changes in the Bax/Bcl2 balance, and limit caspase activation [15,16]. Therefore, enhancing antioxidant capacity and preventing excessive apoptosis in the ovaries and early embryos can maintain ovarian function, promote oocyte development and maturation, and ultimately improve embryo developmental potential and reproductive competence [2].

Recently, there has been increasing interest in the field of regenerative medicine regarding the various types of secretomes secreted by mesenchymal stem cells (MSCs) [17]. The MSC secretome contains various bioactive molecules, including growth factors, cytokines, chemokines, and immunomodulatory factors, which can provide paracrine effects of MSC transplantation without direct cell administration. Furthermore, these cell-free approaches have advantages regarding safety, ease of manufacturing, and standardization [18]. In particular, the MSC secretome or conditioned medium (CM) has been reported to exhibit various biological effects, such as inflammation reduction, oxidative stress alleviation, and promotion of cell survival and tissue microenvironment recovery [3]. Applying these effects to reproductive research, several studies have suggested that MSC-CM can support ovarian function and improve oocyte quality and subsequent embryo development [19,20,21]. However, despite these promising effects, dose optimization and systematic validation are required for the clinical application of CM, considering that optimal conditions may vary depending on the target disease or physiological condition [22].

The amniotic membrane is a fetal-derived tissue that supports a specialized immunological and biological environment throughout gestation [23]. Amniotic membrane-derived mesenchymal stem cells (AMSCs) have recently been recognized as promising cell sources in regenerative medicine [24]. A representative advantage of AMSCs is that the amniotic membrane from which they are isolated can be obtained noninvasively from placental tissue that is normally discarded after delivery, which minimizes ethical concerns and allows a relatively stable cell supply [25]. AMSCs exhibit high proliferative capacity and cellular stability, which are advantageous for mass culture and therapeutic development [26]. Furthermore, they exert anti-inflammatory effects by regulating inflammatory responses and controlling immune cell activity via various secreted factors due to their low immunogenicity and strong immunomodulatory capabilities [27]. AMSCs can differentiate into various mesenchymal tissues, such as bone, cartilage, and fat, while having a low risk of tumorigenesis, offering safety advantages when used as therapeutic cells [28]. Meanwhile, AMSCs exert their effects primarily through paracrine action via secreted factors rather than differentiation, which promotes tissue regeneration, inhibits apoptosis, and regulates the tissue microenvironment in various disease models [29,30,31]. These diverse advantages support the potential development of the AMSC secretome for paracrine-based therapeutic strategies in various disease models.

In this study, we adapted the conventional concept of CM and developed an injectable formulation of the AMSC secretome based on physiological saline instead of cell culture medium to improve clinical applicability by developing safe and practical conditions for its preparation and use. We first assessed the toxicity of human amniotic membrane-derived mesenchymal stem cell-conditioned saline (AMSC-CS) via dose-dependent intravenous administration in female mice. Subsequently, the effect of AMSC-CS on in vitro-fertilized embryo development and redox state-associated molecular markers in ovaries, oocytes, and embryos was evaluated. This study aimed to demonstrate the optimal administration conditions of AMSC-CS for promoting ovarian function and preimplantation embryo development through its antioxidant and anti-apoptotic effects.

## 2. Materials and Methods

### 2.1. Ethics Approval

Human AMSCs were obtained from donors who provided written informed consent before sample collection and were provided by the RNL Regeneration Medicine Research Institute Co., Ltd. under GMP conditions. This study was conducted in accordance with the Declaration of Helsinki and was approved by the Ethics Committee of Biostar Stem Cell Technology (IRB no. 2021-01). All experimental animal procedures were approved by the Institutional Animal Care and Use Committee of Seoul National University (SNU-210518-4-1).

### 2.2. Culture and Characterization of AMSCs

AMSCs were isolated and established using previously described methods [29]. Briefly, cryopreserved AMSCs (1 × 10^6^ cells) were thawed and seeded into T-175 flasks (175 cm^2^) containing RPME-P medium (RNL Regeneration Medicine Research Institute Co., Ltd., Seoul, Republic of Korea) supplemented with 1% antibiotic–antimycotic. The cells were cultured in a humidified incubator at 37 °C and 5% CO_2_. The AMSCs were plastic-adherent under culture conditions and displayed a spindle-shaped, fibroblast-like morphology, consistent with MSC characteristics. The immunophenotypic characterization of the cultured AMSCs was performed using flow cytometry, as previously described [29].

### 2.3. Preparation of AMSC-CS

The AMSCs were cultured until 90% confluency, with medium changes every 2–3 days. Upon reaching the target confluence, the cells were washed twice with phosphate-buffered saline (PBS), and the culture medium was replaced with saline. The cells were then maintained in saline for 3 consecutive days. After incubation, the saline containing the secreted factors was collected and filtered using a 0.22 µm filter, and the obtained medium was designated as conditioned saline. Additional assessments of AMSC viability during saline conditioning, as well as qualitative profiling and sterility testing of AMSC-CS, were performed. The corresponding data are provided in Supplementary Tables S1–S3.

### 2.4. Intravenous Administration of AMSC-CS

Seven-week-old female ICR mice (DBL Inc., Eumseong, Republic of Korea) were housed in the Seoul National University animal facility under conventional conditions. The mice were randomly divided into control and treatment groups: a low-dose group (1 μL/g), a medium-dose group (3 μL/g), and a high-dose group (5 μL/g). The single-dose amount was determined based on the weight of each mouse (1, 3, or 5 μL/g), keeping all injection volumes within accepted technical and animal welfare constraints. The high dose (5 µL/g) was set to the commonly recommended upper limit for intravenous bolus injections in mice (5 mL/kg), while the low dose (1 µL/g) was chosen as a conservative volume within this recommended range [32,33]. The medium dose (3 µL/g) was selected as an intermediate volume between these two bounds to assess potential dose-dependent effects. The administration frequency was set to six times with four-day intervals based on previous research [34]. The control group was administered the same volume of normal saline (0.9%) (JW Pharmaceutical, Seoul, Republic of Korea) as the low-dose group. All animal experiments were conducted using separate cohorts for safety and reproductive assessment. Five mice per cage were housed in an individually ventilated caging system in polysulfone cages. The mice were housed in a controlled environment maintained at a temperature of 21–25 °C and a relative humidity of 40–60% with a 12 h light/dark cycle. Throughout the study period, mice had ad libitum access to a gamma-irradiated, sterilized solid diet (RODENT NIH-41, Zeigler Bros., Inc., Gardners, PA, USA) and autoclaved reverse osmosis (RO) water.

### 2.5. Clinical Observations and Physiological Parameters

Starting from the first dosing day until terminal necropsy, all mice were monitored three times per week for general clinical signs, including appearance, body function, responsiveness to external stimuli, and behavioral features [35]. During the same period, body weight was monitored three times per week. Weekly body weight data (recorded on days 0, 7, 14, 21, and 28) were selected and reported to track the progression of growth and health status. Feed intake was measured on a per-cage basis and reported as the total consumption per cage to assess the overall dietary status of each group.

### 2.6. Gross Necropsy and Organ Examination

All mice were fasted for 3 h and anesthetized with Avertin (2,2,2-tribromoethanol, Aldrich, Milwaukee, WI, USA) to allow for blood collection, followed by humane euthanasia for terminal necropsy. A blinded macroscopic examination was conducted by a qualified veterinarian to detect abnormalities in major organs, including the heart, lungs, liver, spleen, thymus, adrenal glands, kidneys, and ovaries. When macroscopic abnormalities were identified, the affected organs were subjected to histopathological evaluation as a follow-up assessment. Following the gross examination, the absolute weights of the major organs were measured. The relative organ weights were calculated as the ratio of absolute organ weight to terminal body weight and expressed as a percentage to account for variations in individual body weight.

### 2.7. Serum Biochemistry

Serum biochemical analysis was performed to comprehensively evaluate the physiological functions of vital organs and to identify potential systemic metabolic alterations induced by AMSC-CS. Blood samples collected via cardiac puncture were centrifuged at 3000 rpm for 15 min at 4 °C to obtain the serum. Serum levels of aspartate aminotransferase (AST), alanine aminotransferase (ALT), alkaline phosphatase (ALP), total bilirubin (TB), total protein (TP), albumin (ALB), the albumin/globulin ratio (A/G ratio), blood urea nitrogen (BUN), creatinine (CRN), cholesterol (CHOL), triglycerides (TGs), glucose, sodium (Na), potassium (K), chloride (Cl), calcium (Ca), and phosphorus (P) were measured using automated chemistry analyzers (Hitachi 7180; Hitachi, Tokyo, Japan; Fuji dry chemistry analyzer; Fujifilm, Tokyo, Japan; IDEXX Catalyst; IDEXX Laboratories, Westbrook, ME, USA). Electrolytes were measured using an electrolyte analyzer (Easy-Lyte Plus; Medica Corporation, Bedford, MA, USA).

### 2.8. Oocyte Recovery

Superovulation induction and oocyte collection were performed within 7 days after the final administration of AMSC-CS. To induce superovulation, the control and treatment groups received an intraperitoneal injection of 7.5 IU pregnant mare serum gonadotropin, followed 48 h later by a second intraperitoneal injection of 7.5 IU human chorionic gonadotropin. Cumulus–oocyte complexes (COCs) were recovered from the oviductal ampullae 15–17 h after hCG injection and transferred into a droplet of CARD medium (Cosmo Bio Co., Tokyo, Japan). The number of ovulated oocytes for each group was determined by counting the recovered COCs under a stereomicroscope.

### 2.9. In Vitro Fertilization

Mature male mice were euthanized via cervical dislocation, and the caudal epididymides were excised. The caudal epididymal duct was incised, and sperm were dispersed within a CARD medium microdrop (Cosmo Bio Co., Tokyo, Japan). The sperm were incubated for 1 h at 37 °C to allow capacitation. For insemination, COCs were inseminated with the sperm suspension in CARD medium microdrops and incubated for 3 h at 37 °C. Presumptive zygotes were washed and cultured in fresh human tubal fluid (HTF; Cosmo Bio Co., Tokyo, Japan) at 37 °C. After 24 h, embryos that had cleaved to the 2- or 4-cell stage were selected and further cultured in droplets of KSOM medium (Cosmo Bio Co., Tokyo, Japan) for 96 h in a humidified incubator at 37 °C with 5% O_2_ and 5% O_2_.

### 2.10. Embryo Development Evaluation and Total Cell Counts

Embryo development in the control and treatment groups was evaluated using a stereomicroscope by counting embryos reaching the 4-cell, 16-cell, morula, blastocyst, and hatched blastocyst stages. Blastocysts from each group were rinsed with PBS and fixed with 4% (*w*/*v*) paraformaldehyde prepared in PBS. The blastocysts were stained with Hoechst 33342 (5 µg/mL) for 12 min to determine the total cell number. After staining, blastocysts were washed in PBS, placed on glass slides, and covered with coverslips. The total cell number of blastocysts was then assessed using a fluorescence microscope (Nikon Corp., Tokyo, Japan).

### 2.11. Oxidative Stress and Antioxidant Capacity Assays

The levels of hydrogen peroxide (H_2_O_2_) and total antioxidant capacity (TAC) in the ovaries were quantified with OxiSelect™ assay kits (Cell Biolabs, Inc., San Diego, CA, USA) following the manufacturer’s protocol. Briefly, ovarian tissues were homogenized and centrifuged, and the ovarian tissue lysates were collected for subsequent assays, as recommended in the kit protocols. The results of each colorimetric assay were analyzed based on absorbance readings at 490 nm for TAC activity and 570 nm for H_2_O_2_ activity.

### 2.12. Measurement of ROS and GSH Levels

Oocytes and blastocysts were stained for 30 min, respectively, with H2DCFDA (2′,7′-dichlorodihydrofluorescein diacetate) and CellTracker Blue (4-chloromethyl-6,8-difluoro-7-hydroxycoumarin), each diluted in 1% (*w*/*v*) polyvinyl alcohol in phosphate-buffered saline (PVA-PBS). Stained oocytes and blastocysts were washed and transferred to fresh 1% PVA–PBS drops, overlaid with mineral oil. Fluorescence intensity was measured with an epifluorescence microscope (TE2000-S; Nikon, Tokyo, Japan) using filter settings for ROS and GSH detection at 460 and 370 nm, respectively. Quantification was performed with ImageJ v1.52 (National Institutes of Health, Bethesda, MD, USA).

### 2.13. Gene Expression Analysis Using Quantitative Real-Time PCR

Total RNA was extracted from ovaries and blastocysts from each group using the RNAqueous™-Micro Total RNA Isolation Kit (Ambion, Austin, TX, USA). The purity and concentration of RNA were assessed using NanoDrop spectrophotometry (A260/A280 ratio ≥ 2.0; A260/A230 ≥ 2.0). Complementary DNA (cDNA) was synthesized with the Maxime RT premix kit (iNtRON, Seongnam, Republic of Korea). Relative gene expression was analyzed with quantitative real-time PCR using a StepOnePlus Real-Time PCR System (Applied Biosystems, Foster City, CA, USA). Each reaction was prepared in a final volume of 20 μL, consisting of 1 μL of cDNA, 0.4 μL each of forward and reverse primers, 10 μL of SYBR Green Master Mix (Applied Biosystems, Foster City, CA, USA), and 7.2 μL of nuclease-free water (Ambion, Austin, TX, USA). The PCR program began with denaturation at 95 °C for 10 min, followed by 40 amplification cycles at 95 °C for 10 s, 60 °C for 20 s, and 72 °C for 40 s. All samples were analyzed in at least triplicate, and melting-curve analysis was included to confirm amplification specificity. Relative mRNA levels were determined after normalization against 18S rRNA as the housekeeping control. Quantification was based on threshold cycle (Ct) values, which are inversely proportional to transcript abundance; a two-fold dilution of the template is expected to increase Ct by approximately 1 cycle. The relative expression (R) was calculated using the 2^−ΔΔCt^ method. The list of primers used for the analysis is provided in Table 1.

### 2.14. Statistical Analysis

Statistical analyses were performed using GraphPad Prism version 5 (GraphPad; San Diego, CA, USA). When the assumption of normality was met, data were analyzed using one-way ANOVA followed by Tukey’s post hoc test and two-way ANOVA followed by Bonferroni’s post hoc test. When the assumption of normality was not satisfied, data were analyzed using the Kruskal–Wallis test followed by Dunn’s post hoc multiple comparisons test, which accounts for multiple testing. The number of animals used per group in each experiment is provided in the figure legends. Sample sizes were determined with reference to a degrees-of-freedom-based formula for ANOVA in animal studies [36]. The data are expressed as the mean ± standard deviation (SD). Statistical significance was set at *p* < 0.05. Each experiment was repeated at least three times.

## 3. Results

### 3.1. Clinical Observations and Physiological Parameters

Throughout the observation period, no mortality or treatment-related clinical signs were observed in any dose group. All animals appeared healthy with normal posture, respiration, and spontaneous behavior. No abnormalities were detected in the eyes, ears, oral cavity, or external genital regions, and the condition of the skin, coat, and tail was assessed as normal in all groups. There were no significant differences in body weight or average weight gain between the groups during the observation period, regardless of the administered dose (Table 2). Similarly, the daily feed intake was similar in all groups (Table 3).

### 3.2. Gross Necropsy Findings and Organ Evaluation

The gross necropsy results showed no macroscopic lesions in any treatment group. The heart, liver, lungs, spleen, thymus, kidneys, adrenal glands, and ovaries appeared normal upon gross examination. Accordingly, no organs required follow-up histopathological evaluation based on gross necropsy findings. The absolute and relative weights of the organs were similar across all groups, and no dose-related changes were observed (Table 4 and Table 5).

### 3.3. Serum Biochemistry

Serum levels of various biochemical markers, including liver function indicators (AST, ALT, ALP, TBIL, TP, ALB, and A/G ratio), renal function markers (BUN and CREA), lipid profiles (TCHO and TG), glucose, and electrolytes (Na, K, Cl, Ca, and IP), were measured. No significant differences were observed between the groups for any parameter, and no abnormal values were detected in any dose group (Table 6).

### 3.4. Oocyte and Embryo Developmental Competence

Oocyte and embryo developmental competence was evaluated by counting the number of oocytes and embryos and determining developmental rates at sequential preimplantation stages. Embryo developmental rate and total cell number per blastocyst were quantitatively assessed. As described in the Section 2, embryo development was evaluated by counting embryos reaching the 4-cell, 16-cell, morula, blastocyst, and hatched blastocyst stages. In addition, the total cell number per blastocyst was analyzed as a quantitative parameter reflecting blastocyst quality. No adverse effects of treatment were observed on oocyte production, as the number of total ovulated oocytes was comparable between the control and all treated groups. Fertilization rates were significantly higher in the medium- and high-dose groups compared with the low-dose group, although no significant difference was observed relative to the control. Notably, the medium- and high-dose groups exhibited significantly higher cleavage rates than the control and low-dose groups (Table 7). The four-cell development rate was significantly higher in the high-dose group compared with the low-dose group. The 16-cell and morula development rates were lower in the low-dose group compared with the control and higher-dose groups. Blastocyst (BL) development rates were significantly higher in the high-dose group compared with the control, whereas the low- and medium-dose groups showed no significant differences from the control. Hatched blastocyst rates were significantly elevated in the medium- and high-dose groups compared with the control. Total cell numbers in blastocysts were significantly increased in the high-dose group compared with the control (Table 8).

### 3.5. Ovarian Oxidative Status

**Figure 1:**
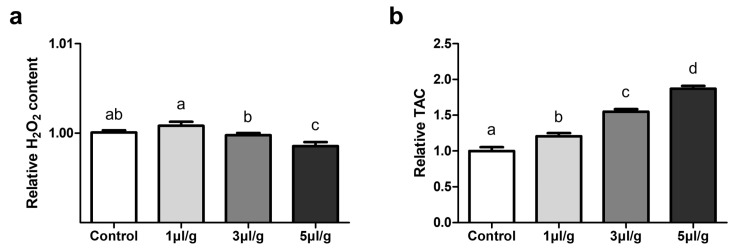
Reactive oxygen species (ROS) and antioxidant contents in ovaries from the groups administered with AMSC-CS. (**a**) ROS (hydrogen peroxide, H_2_O_2_) and (**b**) antioxidants (total antioxidant capacity, TAC). Data are normalized to the average value of the control and presented as the mean ± SD; different superscript letters indicate statistically significant differences among groups (*p* < 0.05). Groups sharing at least one superscript letter are not significantly different (n = 18 biological replicates per group).

Ovarian oxidative status at the tissue level was evaluated by measuring ovarian hydrogen peroxide (H_2_O_2_) content and total antioxidant capacity (TAC). Ovarian H_2_O_2_ levels were significantly reduced in the high-dose group compared with the control and low- and medium-dose groups (Figure 1a). In contrast, TAC was significantly increased in all treated groups, with a trend toward greater enhancement at higher doses, indicating an overall improvement in the ovary’s antioxidant capacity (Figure 1b).

### 3.6. Oocyte Redox Status

**Figure 2:**
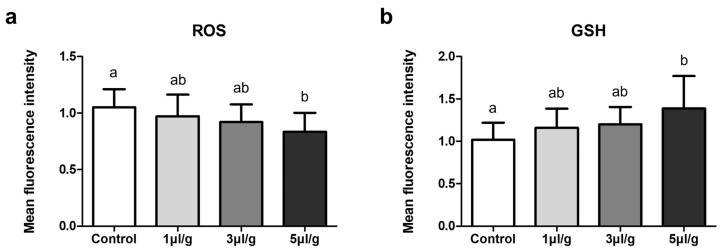
Intracellular reactive oxygen species (ROS) and glutathione (GSH) in oocytes from the groups administered with AMSC-CS. (**a**) ROS and (**b**) GSH levels in oocytes. Experiments were independently repeated at least 3 times (n = 10–15 biological replicates per group). Data are normalized to the average value of the control and presented as the mean ± SD; different superscript letters indicate statistically significant differences among groups (*p* < 0.05). Groups sharing at least one superscript letter are not significantly different.

The cellular redox status of oocytes was assessed using intracellular ROS and GSH staining. The high-dose treatment significantly reduced ROS fluorescence intensity compared with the control group, while the low- and medium-dose groups showed no significant differences (Figure 2a). Conversely, intracellular GSH levels were significantly elevated only in the high-dose group relative to the control, with no changes observed in the lower-dose groups (Figure 2b).

### 3.7. Blastocyst Redox Status

**Figure 3:**
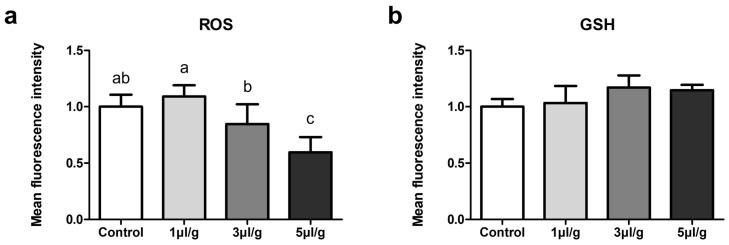
Intracellular reactive oxygen species (ROS) and glutathione (GSH) in blastocysts from the groups administered with AMSC-CS. (**a**) ROS and (**b**) GSH levels in blastocysts. Experiments were independently repeated at least 3 times (n = 10–15 biological replicates per group). Data are normalized to the average value of the control and presented as the mean ± SD; different superscript letters indicate statistically significant differences among groups (*p* < 0.05). Groups sharing at least one superscript letter are not significantly different.

The cellular redox status of blastocysts was assessed using intracellular ROS and GSH staining. The ROS levels were significantly reduced in the high-dose group compared with the control and the low- and medium-dose groups (Figure 3a). In contrast, there were no significant differences in GSH levels between the control and all treated groups (Figure 3b).

### 3.8. Ovarian Antioxidant- and Apoptosis-Related Gene Expression

**Figure 4:**
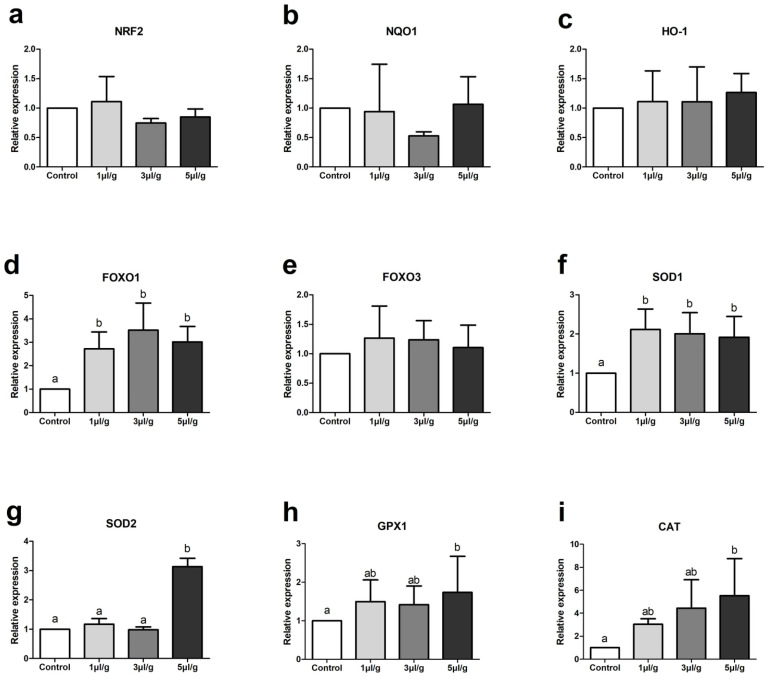
Antioxidant gene expression in ovaries from the groups administered with AMSC-CS. (**a**) *NRF2*, (**b**) *NQO1*, (**c**) *HO-1*, (**d**) *FOXO1*, (**e**) *FOXO3*, (**f**) *SOD1*, (**g**) *SOD2*, (**h**) *GPX1*, and (**i**) *CAT*. Data are normalized to housekeeping gene 18S rRNA and presented as the mean ± SD; different superscript letters indicate statistically significant differences among groups (*p* < 0.05). Groups sharing at least one superscript letter are not significantly different.

**Figure 5:**
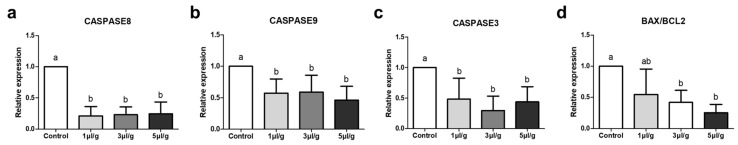
Apoptosis-related gene expression in ovaries from the groups administered with AMSC-CS. (**a**) *CASPASE8*, (**b**) *CASPASE9*, (**c**) *CASPASE3*, and (**d**) *BAX/BCL2* expression ratio. Data are normalized to housekeeping gene 18S rRNA and presented as the mean ± SD; different superscript letters indicate statistically significant differences among groups (*p* < 0.05). Groups sharing at least one superscript letter are not significantly different.

Ovarian expression of antioxidant- and apoptosis-related genes was analyzed to evaluate the effects of AMSC-CS on cellular stress responses at the tissue level. Among transcription factors and key enzymatic antioxidants, *NRF2*, *NQO1*, *HO-1*, and *FOXO3* showed similar expression levels across all groups, with no significant differences compared with the control. In contrast, *FOXO1* expression was significantly increased in the low-, medium-, and high-dose groups relative to the control. *SOD1* expression was also significantly upregulated in all treated groups compared with the control. *SOD2* expression was significantly higher in the high-dose group than in the control and low- and medium-dose groups. Furthermore, *GPX1* and *CAT* expression levels were significantly increased only in the high-dose group compared with the control (Figure 4). The results of apoptosis-related gene expression analysis showed that the *BAX/BCL2* ratio was significantly reduced in the medium- and high-dose groups compared with the control group. *CASPASE3* expression was significantly lower in the low-, medium-, and high-dose groups than in the control. The mRNA levels of *CASPASE8* and *CASPASE9* were significantly decreased in all treatment groups compared with the control (Figure 5).

### 3.9. Ovarian Follicular Development and Maturation-Related Gene Expression

**Figure 6:**
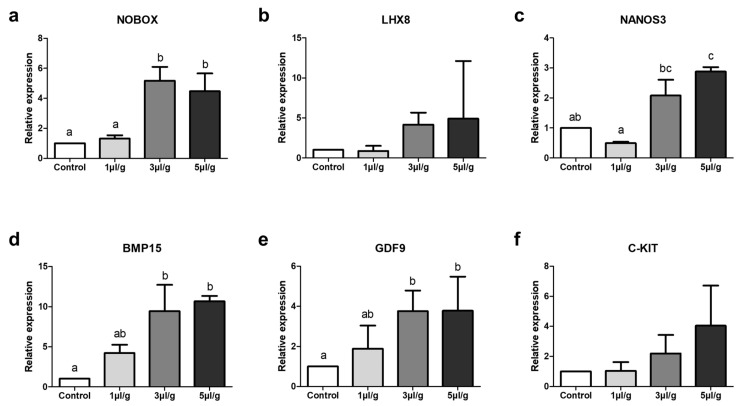
Follicular development and maturation-related gene expression in ovaries from the groups administered with AMSC-CS. (**a**–**c**) Follicular development-related genes: (**a**) *NOBOX*, (**b**) *LHX8*, and (**c**) *NANOS3*; (**d**–**f**) follicular maturation-related genes: (**d**) *BMP15*, (**e**) *GDF9*, and (**f**) *C-KIT*. Data are normalized to housekeeping gene 18S rRNA and presented as the mean ± SD; different superscript letters indicate statistically significant differences among groups (*p* < 0.05). Groups sharing at least one superscript letter are not significantly different.

The expression of genes involved in follicular development and oocyte maturation was examined to assess the impact of AMSC-CS on ovarian developmental competence. Among the genes regulating early follicular development, *NOBOX* expression was significantly increased in the medium- and high-dose groups compared with the control and low-dose groups. *NANOS3* expression was significantly higher in the high-dose group than in the control and low-dose groups, as well as significantly elevated in the medium-dose group compared with the low-dose group. *LHX8* expression showed an increasing trend in the medium- and high-dose groups, but the difference did not reach statistical significance (Figure 6a–c). Among follicular growth- and oocyte maturation-related genes, *BMP15* and *GDF9* expressions were significantly upregulated in the medium- and high-dose groups compared with the control. *C-KIT* expression tended to increase in the medium- and high-dose groups, although the changes were not statistically significant (Figure 6d–f).

### 3.10. Blastocyst Gene Expression

**Figure 7:**
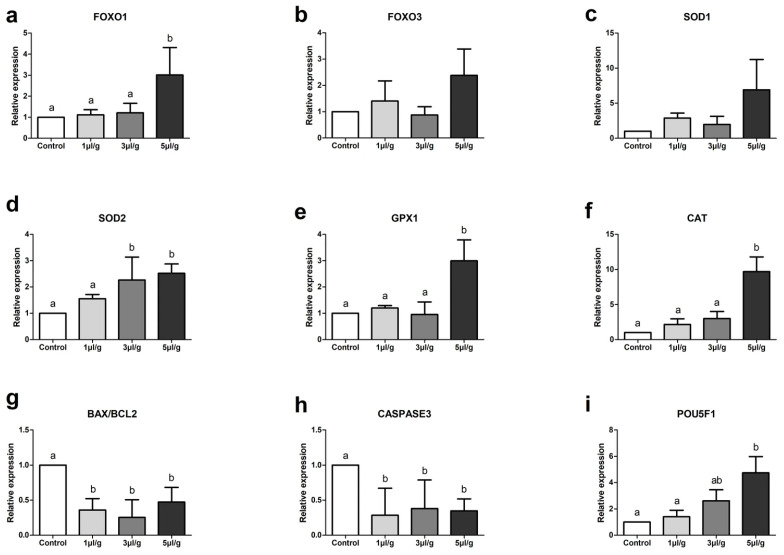
Gene expression in blastocysts from the groups administered with AMSC-CS. (**a**–**f**) antioxidant-related genes: (**a**) *FOXO1*, (**b**) *FOXO3*, (**c**) *SOD1*, (**d**) *SOD2*, (**e**) *GPX1*, and (**f**) *CAT;* (**g**,**h**) apoptosis-related genes: (**g**) *BAX/BCL2* expression ratio and (**h**) *CASPASE3*; and (**i**) *POU5F1*, an embryonic pluripotency-related gene. Data are normalized to housekeeping gene 18S rRNA and presented as the mean ± SD; different superscript letters indicate statistically significant differences among groups (*p* < 0.05). Groups sharing at least one superscript letter are not significantly different.

Gene expression levels associated with antioxidant defense, apoptosis, and pluripotency were measured in blastocysts. Among antioxidant-related genes, *FOXO3* and *SOD1* expression tended to increase in the high-dose group, but this change did not reach statistical significance. *FOXO1* expression was significantly increased in the high-dose group compared with the control and low- and medium-dose groups. *SOD2* expression was significantly upregulated in the medium- and high-dose groups relative to the control. *GPX1* and *CAT* expression levels were significantly elevated in the high-dose group compared with the control and all other treated groups (Figure 7a–f). The *BAX/BCL2* ratio and *CASPASE3* expression were significantly decreased in all treated groups compared with the control (Figure 7g,h). Lastly, the pluripotency marker *POU5F1* expression was significantly higher in the high-dose group compared with the control and low-dose groups (Figure 7i).

## 4. Discussion

This study demonstrates that intravenous administration of AMSC-CS enhances the antioxidant and anti-apoptotic environment at the ovarian and embryonic stages in a dose-dependent manner without causing systemic toxicity, thereby significantly improving follicular development, maturation markers, and in vitro fertilization embryo developmental competence.

AMSC-CS administration did not have an adverse effect on overall health status during the study period. No mortality was observed in any dose group, and no adverse signs related to AMSC-CS administration were identified. AMSC-CS administration did not affect the growth or nutritional status (Table 2 and Table 3). These results suggest that AMSC-CS did not impair systemic status or cause acute or subacute clinical side effects during the study period. No abnormal findings were identified during the gross examination and organ evaluation following the autopsy (Table 4 and Table 5). In addition, the serum chemistry results show that AMSC-CS did not impair major organ function under the conditions of this study (Table 6). Therefore, these clinical observations and findings regarding physiological indicators, serum chemistry, and organ evaluation, as well as the lack of evidence of systemic or target organ toxicity, demonstrate the safety of AMSC-CS within the dosage range established in this study. Considering detailed histopathological assessment was not performed in mice without macroscopic abnormalities, future studies with longer exposure periods or higher cumulative doses may include histopathological evaluation of the liver, kidneys, and ovaries to provide additional tissue-level confirmation of safety.

Next, the control and AMSC-CS groups showed comparable total ovulated oocyte counts and fertilization rates, while cleavage rates after fertilization were increased in the medium- and high-dose AMSC-CS groups (Table 7). The findings indicate that AMSC-CS exhibited a safe profile, with no observed adverse events associated with ovulation, fertilization, or cleavage, irrespective of the administered dose. Follicular development and maturation gene expression was upregulated in the medium- and high-dose groups in mouse ovaries exhibiting normal ovulation (Figure 6). *NOBOX*, *BMP15*, and *GDF9* are molecular markers associated with follicular development and oocyte maturation, and previous studies have demonstrated that their upregulation is correlated with improved ovarian function, enhanced follicular development, and superior oocyte quality [37,38,39]. In addition, a high dose of AMSC-CS significantly upregulated *NANOS3* alongside *NOBOX*, *BMP15*, and *GDF9*, collectively indicating enhanced germ cell survival, oocyte transcriptional activation, and folliculogenesis competence (Figure 6) [40]. In particular, these genes are critically associated with primary ovarian insufficiency (POI), which has reproductive implications and causes infertility [41,42]. In this context, the therapeutic effects of AMSC-CS in reproductive disease models, including POI, need to be validated via further studies to expand its applicability.

AMSC-CS treatment was accompanied by activation of the ovarian antioxidant defense system. *FOXO1*, a key regulator of the FOXO pathway, was significantly upregulated across all treatment groups, with *SOD1* also increased in all dose groups (Figure 4). Specifically, the high-dose group showed significantly increased expression of antioxidant genes *SOD2*, *GPX1*, and *CAT* (Figure 4), which are directly upregulated by *FOXO1* via binding to antioxidant response elements in their promoters, thereby enhancing cellular antioxidant capacity and mitigating oxidative stress [43]. These transcriptional alterations were functionally validated by reduced H_2_O_2_ levels in the high-dose group and by dose-dependent TAC elevation (control < low < medium < high dose) (Figure 1). These results suggest that AMSC-CS may induce regulation of the ovarian redox environment toward an antioxidant-dominant state against oxidative stress, thereby establishing a favorable foundation for oocyte and embryo development at the molecular and physiological levels. This is particularly important considering that oocytes and embryos are highly sensitive to oxidative stress and rely on antioxidants to suppress ROS and maintain cellular homeostasis [2,3,44,45]. Concurrently, gene expression analysis of extrinsic and intrinsic apoptosis pathways showed that AMSC-CS administration significantly reduced the expression of apoptosis-promoting genes in the ovaries (Figure 5). In particular, *CASPASE3*, a key mediator of apoptosis, was significantly reduced at medium and high doses, suggesting an overall attenuation of the caspase cascade and mitochondrial-mediated apoptosis signaling (Figure 5) [46,47]. These results suggest that AMSC-CS improves oocyte quality by creating an antioxidant and anti-apoptotic microenvironment in the ovaries rather than increasing ovulation volume.

The results of IVF clearly demonstrate an improvement in reproductive capacity depending on the dosage of AMSC-CS administration. High doses consistently showed superior effects compared with low doses at all embryonic developmental stages, from the four-cell stage to hatched blastocysts. Later developmental stages (blastocyst formation and hatching rate) in particular exhibited improvements compared with the control group, and the total cell number of blastocysts also increased (Table 8). The total cell number of blastocysts is a widely used indicator of the quality and developmental potential of blastocysts [48]. The increase observed in the high-dose group in this study suggests that AMSC-CS improves the developmental rate as well as indicators related to embryonic quality. Additionally, in the high-dose group, the expression of the pluripotency-related gene *POU5F1* increased in blastocysts, which is consistent with the improvement in inner cell mass quality and may indicate the enhanced developmental potential of IVF embryos (Figure 7) [49,50,51]. The results of ROS/GSH analysis at the oocyte and blastocyst stages presented in Figure 2 and Figure 3 support these developmental results. Although there was no significant difference in GSH levels between groups at the blastocyst stage, the decrease in ROS suggests that the oxidative stress burden on the embryo was reduced [52]. In addition, the changes in gene expression at the blastocyst stage (Figure 7) support an integrated mechanism in which administering high doses of AMSC-CS stabilizes the embryonic development program by enhancing antioxidant and anti-apoptotic effects. In recent years, an increasing number of studies have shown that various forms of stem cell-derived secretions improve the quality of oocytes and embryos, enhance antioxidant capacity, and exhibit anti-apoptotic effects by regulating developmental molecular pathways, which is consistent with the results of this study. For instance, previous work has demonstrated that human adipose MSC-CM reduced oxidative stress and apoptosis in mouse preimplantation embryos, thereby improving their developmental competence in vitro [53]. Consistently, another study showed that human umbilical cord MSC-CM enhanced the maturation and developmental potential of immature human oocytes by modulating apoptotic and stress-related gene expression [54]. In addition, a recent study demonstrated that human amniotic MSC-derived extracellular vesicles improved oocyte maturation and embryonic development in aged mice by enhancing antioxidant defenses and mitochondrial function [55].

Overall, this study’s results suggest that the intravenous administration of AMSC-CS at medium or higher doses can enhance in vitro reproductive capacity without adversely affecting ovulation or fertilization. Collectively, our data suggest a mechanistic interpretation in which AMSC-CS may help improve the ovarian and follicular microenvironment by strengthening antioxidant defense mechanisms and inhibiting the activation of apoptotic pathways. Recent studies have reported that MSC-derived conditioned media or secretome exhibit antioxidant effects in the ovary and other various tissues, primarily via paracrine factors that enhance endogenous antioxidant defenses and alleviate oxidative stress [3,29,53,56,57,58,59,60,61]. Consistent with these findings, the ovarian antioxidant system (including enzymatic defense and GSH-dependent redox regulation) was enhanced after AMSC-CS administration in this study, which may have reduced CASPASE- and BAX/BCL2-mediated apoptosis signaling. Furthermore, the overall upregulation of genes related to follicular development and maturation is consistent with the enhancement of molecular programs that support follicle formation, oocyte growth, and the acquisition of oocyte developmental capacity. From this perspective, the improvement in in vitro-fertilized embryo development and blastocyst quality observed in our experiment may be regarded as a functional indicator of antioxidative and anti-apoptotic conditions extending from the ovary and oocyte to the preimplantation embryonic development stage. These results demonstrate the potential for applying AMSC-CS to assisted reproductive technologies. In this study, the control group received the same injection volume as the low-dose AMSC-CS group, whereas separate volume-matched vehicle controls were not included for the medium- and high-dose groups. The low-dose AMSC-CS demonstrated statistically significant regulations over this volume-matched control in antioxidant contents in ovaries, antioxidant and apoptosis-related gene expression in ovaries, and apoptosis-related gene expression in blastocysts, indicating that the observed effects are attributable to AMSC-CS rather than to the injected saline volume. In addition, despite the differences in injection volume, physiological parameters, including serum biochemistry, did not differ between the groups, suggesting that the variation in injection volume did not induce detectable physiological changes. However, considering a potential influence of the larger saline volume cannot be completely excluded, future studies will include a vehicle control group receiving the maximum injection volume to more rigorously account for any possible volume-related effects. In addition, further in vivo studies are required to confirm whether the beneficial effects of AMSC-CS observed in this study are reproducible regarding implantation, pregnancy maintenance, fertility rates, and the long-term safety of offspring.

A potential limitation of this study is that AMSC-CS was collected using saline instead of serum-free culture medium, which is commonly used for CM preparation. Although standard CM preparation protocols use serum-free media to induce cellular starvation for secretome production [62,63], serum-free media still provide a basal environment that supports cell maintenance. Physiological saline lacks nutrients and growth factors, and its use as a conditioning solution may therefore affect cellular viability, secretory activity, and total secretome yield differently from conventional serum-free CM preparation. In addition, prolonged exposure to saline may increase cellular stress. In this regard, reduced cellular viability during saline conditioning should be considered when interpreting AMSC-CS. However, reduced cellular viability during saline conditioning does not necessarily preclude the biological relevance of AMSC-CS. Previous studies on MSC preconditioning and apoptotic MSC-derived products suggest that MSC-derived secretomes or cell-free products can retain biological activity even when parental MSCs are exposed to non-standard, viability-reducing conditions or undergo apoptosis-associated processes. In particular, conditions or preconditioning approaches such as hypoxia, nutrient deprivation, and apoptosis-associated processes have been reported to affect MSC secretory profiles and to generate soluble or vesicular MSC-derived products with biological activities that may contribute to therapeutic effects [64,65,66]. Although AMSC-CS was not designed or characterized as an apoptotic vesicle preparation, these studies support the broader concept that reduced parental MSC viability does not automatically abolish the biological activity of MSC-derived cell-free products. Although MSC viability may decrease during saline conditioning, this should be distinguished from the safety profile of the final cell-free AMSC-CS preparation. In this study, we evaluated the basic safety and reproductive efficacy of AMSC-CS prepared using saline and did not observe overt toxicity or significant adverse reactions under the tested conditions. Furthermore, MSC-associated factors previously reported to be related to pro-angiogenic, anti-apoptotic, and immunomodulatory activities [64,67,68,69] were detected in AMSC-CS in this study, suggesting that the final preparation contained detectable proteinaceous bioactive components despite the nutritionally limited conditioning environment. While this study aimed to evaluate the safety and efficacy of AMSC-CS, future studies comparing AMSC-CS with CM prepared in serum-free culture media would be useful to clarify how saline conditioning affects secretome composition, cellular status during conditioning, and biological activity of the final cell-free preparation. Nevertheless, as a saline-based formulation, AMSC-CS can offer several advantages. First, the results indicate a direct effect of the AMSC secretome through minimization of exogenous components present in the cell culture medium, such as amino acids, vitamins, and residual growth factors, thereby providing clearer insights into the underlying mechanisms. Second, the simplified composition can reduce unnecessary background noise caused by complex medium components, which can improve the analytical accuracy of subsequent analyses, including proteomics and cytokine profiling. Furthermore, there are still challenges in the clinical application of stem cell-derived CMs due to the lack of standardized and reproducible manufacturing protocols [70]. From a practical perspective, saline-based formulations are particularly suitable for clinical use as they ensure reproducibility through compositional transparency and help establish robust quality standards for injectable production. The AMSC-CS strategy aims to comprehensively improve both experimental purity and clinical scalability and is expected to have broad utility in future assisted reproductive research and applications.

## 5. Conclusions

The intravenous administration of AMSC-CS exhibited no clinically apparent adverse effects or dose-dependent toxicity and did not adversely affect ovulation or fertilization at any tested dose. AMSC-CS at medium-to-high doses enhanced the antioxidant and anti-apoptotic environment within mouse ovaries, oocytes, and embryos in a dose-dependent manner, leading to improved follicular development and maturation, as well as enhanced in vitro-fertilized embryo development. As a cell-free therapeutic, AMSC-CS has potential for development as an injectable formulation and for use in clinical and industrial applications. It may also provide practical efficiencies in translational research and serve as a novel therapeutic and research platform in assisted reproduction and broader reproductive research.

## Figures and Tables

**Table 1:** List of primers and sequences used for quantitative reverse transcription–polymerase chain reaction in mouse ovaries. F, forward primer; R, reverse primer.

Gene	GenBank Accession No.	Sequence
*18S rRNA*	NR_003278.3	F: ACCGCGGTTCTATTTTGTTG
		R: CCCTCTTAATCATGGCCTCA
*BAX*	NM_007527.3	F: ACCAAGAAGCTGAGCGAGTG
		R: TGCAGCTCCATATTGCTGTC
*BCL2*	NM_009741.5	F: ATGATAACCGGGAGATCGTG
		R: AGCCCCTCTGTGACAGCTTA
*CASPASE 9*	NM_001277932.1	F: GATGCTGTCCCCTATCAGGA
		R: AAGTCCCTTTCGCAGAAACA
*CASPASE 8*	NM_001080126.1	F: CTTCGAGCAACAGAACCACA
		R: GCAGAAAGTCTGCCTCATCC
*CASPASE 3*	NM_001284409.1	F: TGTCATCTCGCTCTGGTACG
		R: ATTTCAGGCCCATGAATGTC
*NRF2*	NM_010902.4	F: CTCGCTGGAAAAAGAAGTGG
		R: GGAGAGGATGCTGCTGAAAG
*NQO1*	NM_008706.5	F: GGATGGAAGAAACGTCTGGA
		R: GGCTGCTTGGAGCAAAATAG
*HO-1*	NM_010442.2	F: TGCTCGAATGAACACTCTGG
		R: GAAGGCGGTCTTAGCCTCTT
*FOXO1*	NM_019739.3	F: ACATTTCGTCCTCGAACCAG
		R: CAGGTCATCCTGCTCTGTCA
*FOXO3*	NM_019740.3	F: ATGGGAGCTTGGAATGTGAC
		R: TTAAAATCCAACCCGTCAGC
*SOD1*	NM_011434.2	F: GGCAAAGGTGGAAATGAAGA
		R: AATCCCAATCACTCCACAGG
*SOD2*	NM_013671.3	F: CTGTCTTCAGCCACACCAGA
		R: CTGCTCTTCCAAAGGTCCTG
*GPX1*	NM_008160.6	F: CCGACCCCAAGTACATCATT
		R: CCCACCAGGAACTTCTCAAA
*CAT*	NM_009804.2	F: TTGACAGAGAGCGGATTCCT
		R: TCTGGTGATATCGTGGGTGA
*NOBOX*	NM_130869.3	F: GCCAACCTTCCTCTTCCTCT
		R: GTCCCTCATTGGAGGCAGTA
*LHX8*	NM_010713.2	F: CATGGATGCTCACCAACAAC
		R: CATTGGATGGGGTAACAAGG
*NANOS3*	NM_001357361.1	F: CCAAGCCAAGTTCAGAAAGC
		R: TTCAGGAGCTGCAGAGGATT
*BMP15*	NM_009757.5	F: CTCCTGCCATGTGGAAACTT
		R: GGGAGAAGGCTTTGAGGAAC
*GDF9*	NM_008110.2	F: TGGAACACTTGCTCAAATCG
		R: GAAGGAGGGAGGTCACATCA
*C-KIT*	NM_001122733.1	F: TCATCGAGTGTGATGGGAAA
		R: CACGTTTTTGATGGTGATGC
*POU5F1*	NM_013633.3	F: AGCACGAGTGGAAAGCAACT
		R: TTCATGTCCTGGGACTCCTC

**Table 2:** Changes in body weight of mice administered AMSC-CS. Values are expressed as means ± SD (n = 10 biological replicates per group). No statistically significant differences were found between values within the same column (*p* > 0.05). Day 0: day of experimental animal delivery; day 28: day of autopsy.

Group	Body Weight (g)					Weight Gain (g) on Day 28
	Day 0	Day 7	Day 14	Day 21	Day 28	
Control	24.21 ± 0.73	27.11 ± 1.11	28.35 ± 1.17	29.94 ± 1.89	32.69 ± 2.48	8.48 ± 2.08
Low	23.62 ± 1.03	26.81 ± 0.88	27.63 ± 1.29	29.01 ± 0.93	31.16 ± 1.59	7.54 ± 1.67
Medium	23.99 ± 1.09	27.83 ± 1.14	28.88 ± 1.49	30.41 ± 1.49	32.63 ± 1.82	8.65 ± 1.83
High	23.92 ± 0.21	27.23 ± 1.09	27.70 ± 0.96	29.08 ± 1.29	31.68 ± 2.12	7.76 ± 2.16

**Table 3:** Food intake of mice administered AMSC-CS. Values are expressed as means ± SD (n = 10 biological replicates per group). No statistically significant differences were found between values within the same row (*p* > 0.05).

	Control	Low	Medium	High
Daily feed intake (g)	23.09 ± 1.18	22.36 ± 1.36	22.35 ± 2.80	20.55 ± 2.14

**Table 4:** Absolute organ weights (g) of mice administered AMSC-CS. Values are expressed as means ± SD (n = 10 biological replicates per group). No statistically significant differences were found between values within the same row (*p* > 0.05).

	Control	Low	Medium	High
Heart	0.16 ± 0.02	0.15 ± 0.01	0.15 ± 0.02	0.14 ± 0.02
Liver	1.32 ± 0.11	1.30 ± 0.12	1.40 ± 0.39	1.36 ± 0.25
Lung	0.20 ± 0.02	0.22 ± 0.01	0.21 ± 0.01	0.21 ± 0.02
Spleen	0.12 ± 0.01	0.13 ± 0.02	0.12 ± 0.01	0.13 ± 0.02
Thymus	0.08 ± 0.01	0.08 ± 0.01	0.08 ± 0.01	0.08 ± 0.01
Kidney	0.34 ± 0.01	0.33 ± 0.02	0.33 ± 0.02	0.32 ± 0.03
Adrenal	0.01 ± 0.001	0.01 ± 0.001	0.01 ± 0.002	0.01 ± 0.002
Ovary	0.02 ± 0.003	0.02 ± 0.005	0.01 ± 0.003	0.01 ± 0.005

**Table 5:** Relative organ weights (%) of mice administered AMSC-CS. Values are expressed as means ± SD (n = 10 biological replicates per group). No statistically significant differences were found between values within the same row (*p* > 0.05).

	Control	Low	Medium	High
Heart	0.50 ± 0.09	0.49 ± 0.05	0.46 ± 0.08	0.45 ± 0.06
Liver	4.05 ± 0.30	4.15 ± 0.30	4.27 ± 1.03	4.26 ± 0.64
Lung	0.62 ± 0.09	0.71 ± 0.05	0.66 ± 0.03	0.67 ± 0.09
Spleen	0.38 ± 0.04	0.43 ± 0.07	0.38 ± 0.04	0.41 ± 0.08
Thymus	0.25 ± 0.03	0.25 ± 0.05	0.24 ± 0.04	0.26 ± 0.05
Kidney	1.03 ± 0.08	1.07 ± 0.06	1.01 ± 0.06	1.02 ± 0.08
Adrenal	0.03 ± 0.01	0.04 ± 0.01	0.03 ± 0.01	0.03 ± 0.01
Ovary	0.05 ± 0.01	0.05 ± 0.02	0.04 ± 0.01	0.04 ± 0.02

**Table 6:** Serum chemical analysis of mice administered AMSC-CS. Values are expressed as means ± SD (n = 10 biological replicates per group). No statistically significant differences were found between values within the same row (*p* > 0.05). Abbreviations: AST, aspartate aminotransferase; ALP, alkaline phosphatase; BUN, blood urea nitrogen; CREA, creatinine; GLU, glucose; TBIL, total bilirubin; ALB, albumin; TP, total protein; TCHO, total cholesterol; Ca, calcium; IP, inorganic phosphorus; TG, triglyceride; ALT, alanine aminotransferase; Na, sodium; K, potassium; Cl, chloride; A/G ratio, albumin–globulin ratio.

	Control	Low	Medium	High
AST (U/L)	82.00 ± 20.78	76.67 ± 7.23	69.33 ± 10.69	63.33 ± 11.68
ALP (U/L)	77.00 ± 9.64	90.33 ±9.07	79.67 ± 17.62	95.00 ± 14.00
BUN (mg/dL)	16.83 ± 2.23	18.27 ± 1.79	22.43 ± 7.94	19.70 ± 1.90
CREA (mg/dL)	0.42 ± 0.06	0.42 ± 0.02	0.44 ± 0.08	0.44 ± 0.06
GLU (mg/dL)	250.0 ± 39.95	264.7 ± 35.00	262.3 ± 62.08	212.0 ± 20.07
TBIL (mg/dL)	0.09 ± 0.02	0.07 ± 0.01	0.08 ± 0.03	0.06 ± 0.01
ALB (g/dL)	3.07 ± 0.06	3.01 ± 0.17	3.11 ± 0.06	3.17 ± 0.27
TP (g/dL)	4.81 ± 0.02	4.74 ± 0.23	4.89 ± 0.16	4.98 ± 0.42
TCHO (mg/dL)	84.0 ± 6.0	109.7 ± 10.21	116.0 ± 15.72	113.3 ± 22.5
Ca (mg/dL)	8.57 ± 0.11	8.57 ± 0.15	8.67 ± 0.15	8.77 ± 0.40
IP (mg/dL)	7.10 ± 0.17	6.73 ± 0.47	6.70 ± 0.85	6.73 ± 0.65
TG (mg/dL)	80.7 ± 29.14	66.0 ± 15.87	74.3 ± 7.50	83.0 ± 28.69
ALT (U/L)	40.00 ± 10.39	33.67 ± 4.04	37.00 ± 9.64	30.67 ± 4.93
Na (mEq/L)	144.4 ± 0.8	143.8 ± 0.8	146.1 ± 1.8	146.1 ± 1.2
K (mEq/L)	4.95 ± 0.18	4.59 ± 0.32	4.50 ± 0.19	4.41 ± 0.13
Cl (mEq/L)	114.3 ± 0.9	112.8 ± 0.4	115.8 ± 2.9	115.0 ± 1.0
A/G ratio	1.77 ± 0.12	1.75 ± 0.09	1.75 ± 0.06	1.76 ± 0.02

**Table 7:** Quantitative assessment of ovulation, fertilization, and early cleavage outcomes after AMSC-CS administration. Experiments were independently repeated at least 3 times (n = 6 biological replicates per group in each experiment). Data are presented as mean ± SD. Different superscript letters indicate statistically significant differences among groups (*p* < 0.05). Groups sharing at least one superscript letter are not significantly different.

Group	Number of Oocytes	Number of Fertilized Embryos	Fertilization Rate (%)	Cleavage Rate (%)
Control	41.83 ± 8.49	35.63 ± 9.43	84.09 ± 4.95 ^ab^	56.23 ± 6.47 ^a^
Low	42.90 ± 9.48	32.10 ± 8.46	74.76 ± 8.26 ^a^	45.37 ± 6.45 ^a^
Medium	38.40 ± 6.51	32.27 ± 3.07	85.06 ± 6.44 ^b^	73.01 ± 3.03 ^b^
High	31.57 ± 5.06	28.20 ± 5.02	89.06 ± 2.45 ^b^	67.51 ± 2.09 ^b^

**Table 8:** Quantitative assessment of in vitro-fertilized embryo developmental outcomes after AMSC-CS administration. Experiments were independently repeated at least 3 times (n = 6 biological replicates per group in each experiment). Data are presented as mean ± SD. Different superscript letters indicate statistically significant differences among groups (*p* < 0.05). Groups sharing at least one superscript letter are not significantly different.

Group	Embryo Developmental Rate (%) to					Total Cell Number per Blastocyst (n)
	4-Cell	16-Cell	Morula	Blastocyst	Hatched Blastocyst	
Control	78.68 ± 13.11 ^ab^	62.60 ± 15.24 ^b^	61.84 ± 15.66 ^b^	44.35 ± 12.34 ^ab^	20.21 ± 8.70 ^a^	59.00 ± 2.64 ^a^
Low	63.18 ± 7.10 ^a^	41.50 ± 2.42 ^a^	40.00 ± 0.63 ^a^	30.53 ± 7.16 ^a^	24.45 ± 4.98 ^ab^	57.75 ± 8.26 ^a^
Medium	77.66 ± 18.32 ^ab^	58.34 ± 1.52 ^b^	55.84 ± 11.26 ^b^	48.40 ± 9.90 ^bc^	30.10 ± 8.54 ^bc^	70.40 ± 9.01 ^ab^
High	88.12 ± 7.34 ^b^	67.91 ± 9.90 ^b^	65.33 ± 9.39 ^b^	58.38 ± 12.54 ^c^	36.63 ± 8.03 ^c^	77.88 ± 7.18 ^b^

## Data Availability

The original contributions presented in this study are included in the article/Supplementary Material. Further inquiries can be directed to the corresponding author.

## Supplementary Materials

The following supporting information can be downloaded at: https://www.mdpi.com/article/10.3390/biomedicines14071522/s1, Table S1: Approximate viability of AMSCs during saline conditioning at the indicated time points; Table S2: Cytokines, chemokines, and growth factors qualitatively detected in AMSC-CS; Table S3: Sterility, mycoplasma, and viral marker testing results for AMSC-CS.
