# Supplementary material for: Human Amniotic Membrane-Derived Mesenchymal Stem Cell-Conditioned Saline as an Injectable Formulation Improves Ovarian Antioxidant Status and Preimplantation Embryo Development
Source: Biomedicines. 2026 Jul 7;14(7):1522. doi: 10.3390/biomedicines14071522 (PMC13405739; doi:10.3390/biomedicines14071522)
Supplement: Supplementary file 1 [file biomedicines-14-01522-s001.zip › biomedicines-4338267-supplementary.pdf]

Supplementary Methods

Cell viability assessment

AMSC viability during saline conditioning was assessed using trypan blue exclusion and an automated cell counter. The saline conditioning procedure was performed as described in the Materials and Methods section. Briefly, after replacement of the culture medium with sterile physiological saline, AMSCs were maintained for 3 consecutive days at 37°C in a humidified CO_2 incubator. For viability assessment, cells were collected at 0, 8, 24, 36, and 72 h during the saline conditioning period and resuspended to obtain a uniform single-cell suspension. An aliquot of the cell suspension was mixed with 0.4% trypan blue solution, loaded into a counting chamber slide, and analyzed according to the manufacturer's instructions.

Qualitative Luminex profiling

AMSC-CS samples were analyzed using a Human Luminex Discovery Assay platform (R&D Systems, Minneapolis, MN, USA) according to the manufacturer's instructions for multiplex bead-based immunoassays. This analysis was performed as qualitative profiling to confirm the presence of cytokines, chemokines, and growth factors in the conditioned saline preparation.

Sterility, mycoplasma, and viral marker testing

Sterility testing was performed using the membrane filtration method according to the procedures described in the Korean Pharmacopoeia and USP <71>. Samples were filtered through a sterile membrane filter and incubated in appropriate culture media under the recommended conditions for the detection of microbial contamination. Mycoplasma contamination was evaluated using the MycoAlert™ Mycoplasma Detection Assay kit (Lonza, Basel, Switzerland) according to the manufacturer's instructions. This assay is designed as a qualitative test based on the ratio between pre- and post-substrate luminescence readings, with ratios below the manufacturer-defined cutoff interpreted as negative for mycoplasma contamination. Viral marker testing was performed using commercially available diagnostic kits (Abbott Rapid Diagnostics, USA) for human immunodeficiency virus (HIV), hepatitis B surface antigen (HBsAg), and hepatitis C virus (HCV), and the results were interpreted according to the manufacturer's instructions.

Supplementary Results

Cell viability during saline conditioning

AMSCs showed a time-dependent decrease in viability during saline conditioning. Approximate cell viability was greater than 90% at 0 h, 60% at 8 h, 40% at 24 h, 20% at 36 h, and less than 10% at 72 h (Supplementary Table S1).

Table S1. Approximate viability of AMSCs during saline conditioning at the indicated time points.

Time point	Approximate viability (%)
0 h	>90%
8 h	60%
24 h	40%
36 h	20%
72 h	<10%

Qualitative cytokine and growth factor profiling

Qualitative Luminex profiling confirmed the presence of multiple cytokines, chemokines, and growth factors associated with MSC secretomes. The list of detected analytes is provided in Supplementary Table S2.

Table S2. Cytokines, chemokines, and growth factors qualitatively detected in AMSC-CS.

Detected analytes	
IL-6	TRAIL/TNFSF10
PDGF-BB	IGFBP-1
CCL-1	Adiponectin/Acrp30
Pro-collagen I alpha 1	GM-CSF
CXCL12/SDF-1 alpha	bFGF
IFN-beta	IL-8/CXCL8
EGF	Leptin/OB
VEGF-A	MIF
SCF/c-kit Ligand	Fibronectin
IFN-gamma	HGF
BMP-7	BMP-4
LIF	GDNF

Sterility, mycoplasma, and viral marker testing results

No microbial growth, mycoplasma contamination, or viral markers were detected in the tested AMSC-CS preparation (Supplementary Table S3).

Table S3. Sterility, mycoplasma, and viral marker testing results for AMSC-CS.

Test item	Method or marker	Result
Sterility	Membrane filtration	Negative
Mycoplasma	MycoAlert™ mycoplasma detection assay	Negative
Viral marker testing	HIV	Negative
Viral marker testing	HBsAg	Negative
Viral marker testing	HCV	Negative
